# Investigating the association between characteristics of local crisis care systems and service use in an English national survey

**DOI:** 10.1192/bjo.2023.595

**Published:** 2023-11-03

**Authors:** Antonio Rojas-García, Christian Dalton-Locke, Luke Sheridan Rains, Ceri Dare, Cedric Ginestet, Una Foye, Kathleen Kelly, Sabine Landau, Chris Lynch, Paul McCrone, Shilpa Nairi, Karen Newbigging, Patrick Nyikavaranda, David Osborn, Karen Persaud, Nick Sevdalis, Martin Stefan, Ruth Stuart, Alan Simpson, Sonia Johnson, Brynmor Lloyd-Evans

**Affiliations:** NIHR Mental Health Policy Research Unit, Division of Psychiatry, University College London, UK; and Department of Psychiatry, University of Granada, Spain; Division of Psychiatry, University College London, UK; NIHR Mental Health Policy Research Unit, Division of Psychiatry, University College London, UK; Department of Biostatistics and Health Informatics, Institute of Psychiatry, Psychology, and Neuroscience, King's College London, UK; NIHR Mental Health Policy Research Unit, Institute of Psychiatry, Psychology, and Neuroscience, King's College London, UK; Oxford Health NHS Foundation Trust, Oxford, UK; NIHR Mental Health Policy Research Unit, Department of Biostatistics and Health Informatics, Institute of Psychiatry, Psychology, and Neuroscience, King's College London, UK; NIHR Mental Health Policy Research Unit, Institute for Lifecourse Development, University of Greenwich, UK; Camden and Islington NHS Foundation Trust, London, UK; Department of Psychiatry, University of Oxford, UK; NIHR Mental Health Policy Research Unit, Division of Psychiatry, University College London, UK; and Department of Primary Care and Public Health, Brighton & Sussex Medical School, University of Sussex, UK; Department of Psychiatry, University of Granada, Spain; and Camden and Islington NHS Foundation Trust, London, UK; Centre for Implementation Science, Health Service and Population Research Department, Institute of Psychiatry, Psychology, and Neuroscience, King's College London, UK; and NUS Centre for Behavioural & Implementation Science Interventions, Singapore; Mental Health Addictions and Intellectual Disability Directorate, Te Whatu Ora (Southern), Dunedin, New Zealand; NIHR Mental Health Policy Research Unit, Division of Psychiatry, University College London, UK; and Camden and Islington NHS Foundation Trust, London, UK

**Keywords:** Mental health services, crisis care models and systems, psychiatric admissions, psychiatric detentions, England

## Abstract

**Background:**

In England, a range of mental health crisis care models and approaches to organising crisis care systems have been implemented, but characteristics associated with their effectiveness are poorly understood.

**Aims:**

To (a) develop a typology of catchment area mental health crisis care systems and (b) investigate how crisis care service models and system characteristics relate to psychiatric hospital admissions and detentions.

**Method:**

Crisis systems data were obtained from a 2019 English national survey. Latent class analyses were conducted to identify discernible typologies, and mixed-effects negative binomial regression models were fitted to explore associations between crisis care models and admissions and detention rates, obtained from nationally reported data.

**Results:**

No clear typology of catchment area crisis care systems emerged. Regression models suggested that provision of a crisis telephone service within the local crisis system was associated with a 11.6% lower admissions rate and 15.3% lower detention rate. Provision of a crisis cafe was associated with a 7.8% lower admission rates. The provision of a crisis assessment team separate from the crisis resolution and home treatment service was associated with a 12.8% higher admission rate.

**Conclusions:**

The configuration of crisis care systems varies considerably in England, but we could not derive a typology that convincingly categorised crisis care systems. Our results suggest that a crisis phone line and a crisis cafe may be associated with lower admission rates. However, our findings suggest crisis assessment teams, separate from home treatment teams, may not be associated with reductions in admission and detentions.

The development and implementation of mental health services that effectively help to avoid unnecessary acute hospital admissions has been a fundamental objective of researchers and policy makers internationally in recent decades.^1,2^ Widespread dissatisfaction with in-patient care, reports that some patients are traumatised or retraumatised by admissions, lack of clarity regarding what is achieved therapeutically and high costs are among the concerns about in-patient care; rising rates of compulsory detention in the UK and elsewhere^2^ make the need to seek alternatives still more pressing, as compulsory detention is often associated with coercion and contrasts with the principles of consent and collaboration usually seen as essential to good care.^3,4^ Consequently, innovative community crisis care models have emerged in the UK and internationally over several decades, to provide alternatives to in-patient care and improve assessment and triage of mental health crises.^2,5^

## Background

Three community crisis service models are relatively well-established in England and internationally: (a) crisis resolution and home treatment teams, which provide rapid assessment and intensive home treatment usually over a few days or weeks;^6^ (b) acute day units, which provide intensive treatment in settings where patients attend for several hours a day for a mixture of individual and group-based activities and therapeutic interventions;^7^ and (c) crisis houses, which provide non-hospital residential crisis care services.^8^ These three models all have a research base (crisis resolution and home treatment teams,^9,10^ acute day units,^11,12^ crisis houses^13,14^) suggesting that, if implemented well, they can be an acceptable and comparably effective alternative to hospital admission for at least some patients in mental health crisis.

In the UK, the past decade has seen the further development of models intended to reduce pressures on emergency and in-patient mental health services, for which evidence is largely lacking.^8,9,11^ Some newer models, including crisis cafes, street triage teams and crisis assessment services that are distinct from home treatment services, have proliferated, whereas others such as acute day units have decreased.^2^ This has led to a substantial heterogeneity in local crisis care systems both in terms of the types of crisis service available and the ways in which the system as a whole is organised. Thus far, we know of no research investigating which types of service and which ways of organising the system appear most successful in terms of the key aim of avoiding admissions, especially compulsory detentions.

This study therefore involves two main objectives: (a) to determine the types of mental health crisis care systems at the local area commissioning level (in 2019 in England, these were called clinical commissioning groups (CCGs)), and (b) to explore what characteristics of the crisis care systems are associated with mental health hospital admissions and detentions. The second objective was split into two specific objectives focused on (a) whether three theoretically derived variables describing crisis care systems (access, choice and integration) are associated with admissions and detentions, and (b) exploring if any data-derived variable for the presence of specific service models and system characteristics are associated with admissions and detentions.

## Method

The study uses data from a cross-sectional national survey of mental health crisis care systems in England. Managers of crisis resolution teams (CRTs) were asked to provide information about services for adults within the local catchment area crisis care system (CCG). Data were collected between April 2019 and December 2019, and referred to the service configuration as provided at the beginning of April 2019. CRT managers were the preferred survey respondents, as they tend to be familiar with crisis services in their area. Additional respondents were approached where necessary to gain complete data about services within a local area, and respondents and National Health Service (NHS) Trust leads were invited to check the collected data for accuracy. Descriptive results from this survey has already been published.^2^

### Measures

#### Crisis care system characteristics

Data from the national survey in 2019 were originally collected at the level of the local CRT catchment area (CCG).^2^ The national survey included items on the catchment area of the CRT and the CCG that commissioned the service. This information was used to produce variables at the CCG level. Where there were multiple CRTs for a single CCG, the data were combined. Please see supplementary material (Supplementary Appendix 1 available at https://doi.org/10.1192/bjo.2023.595) for further detail on how data were combined to produce variables at the CCG level.

The variables from the national survey of crisis care in England that were used to characterise local crisis care systems are presented in Table 1. These binary variables captured information about system characteristics and the presence or absence of service models across 195 CCGs. The service model variables capture whether various crisis services were present within the catchment area, including as crisis assessment/single point of access services, crisis phone line, psychiatric decision units, crisis cafes, and police and ambulance street triage services. The system characteristics variables provided information about the organisation and delivery of mental healthcare, including joint management at the crisis care team level, shared staffing, voluntary sector involvement and peer-led services.

**Table 1:** Crisis care system variables

Variable	Description
Service models	
Crisis resolution teams	Multidisciplinary teams that provide rapid assessment and intensive home treatment for a limited period during a crisis
Crisis assessment/single point of access services	A service that provides rapid response crisis assessment in a community setting but does not also provide home treatment for acute mental health problems
Crisis phone line	A service that provides crisis response and triage via telephone only, without face-to-face contact (separate from the crisis resolution and home treatment team or crisis assessment team)
Psychiatric decision units	A dedicated space (separate from an accident and emergency department/psychiatric ward/psychiatric liaison team) in which assessment can be conducted and treatment plans developed for patients in mental health crisis who are accessing emergency services. People may typically stay for up to 24–48 h
Triage wards	In-patient psychiatric wards that admit patients for briefer stays than are usual on acute psychiatric wards. Usually these do not admit people compulsorily detained under mental health legislation, and they work closely with local community crisis services to avoid the need to transfer to an acute psychiatric ward
Crisis cafes	A community-based service that provides out of hours assessment and immediate support for people in mental health crisis, intended to reduce pressure on accident and emergency departments
Police street triage services	A team where mental health staff work jointly with police services to arrange appropriate assessment and support for people with mental health problems who come to the attention of the police
Ambulance street triage services	A team where mental health staff work jointly ambulance services to arrange appropriate assessment and support for people with mental health problems who come to the attention of the ambulance service
Crisis houses	A time-limited, non-hospital residential service for people in mental health crisis
Acute day units	A non-residential day hospital/crisis day service providing support, activity and therapeutic groups for people in mental health crisis
Crisis family placements	A service involving families offering short-term crisis foster placements in their family home for people in mental health crisis, supported by local crisis services
System characteristics	
Integrated management	Presence of joint management at team level between any crisis services
Integrated staffing	Presence of shared staffing between any crisis services
Voluntary sector provision	Involvement of voluntary sector organisation in providing any crisis service
Peer-led services	Presence of peer-led services in delivering any crisis service

In addition, three further theoretically derived variables were considered: access, choice and integration. These variables were constructed from the service models variables and are intended to measure three specific concepts: (a) access (scores range from 0 to 8) was based on the information about 24/7 access to crisis services, accepting self-referrals, accepting referral from other agencies and providing a 24/7 CRT response; (b) choice (scores range from 0 to 6) of crisis service was created by using the information on the availability of crisis cafes, crisis houses, acute day units and crisis family placements; and (c) integration (scores range from 0 to 6) was produced with information on mental health crisis services, acute wards and emergency services to capture integrative process on crisis care. Please see Supplementary Appendix 1 for detailed definitions of these variables.

### Outcome variables

To complement the information collected in the survey, Public Health England (PHE) Fingertips data were accessed^15^ to obtain information on hospital admissions and detention at the CCG level. PHE Fingertips is a publicly available health data-set on service use. The two outcome variables were mental health admission and detention rates. The admissions variable was defined as the number of hospital provider spells in secondary mental health services expressed as a rate per 100 000 person-years within the catchment area. The mental health detention rate variable was also expressed per 100 000 person-years and defined as the number of people detained under the Mental Health Act 1983 (adult mental health and children and young people services only). The reporting period for both outcomes variables was between 1 April 2018 and 31 March 2019. As detentions were reported quarterly, 3-month totals were added together for a cumulative total for a year. The counts for mental health admissions and detentions were calculated by using the population size by CCG in 2018.^16^

### Covariates/putative confounders used in analyses

Potential covariates were also obtained for the period 2018–2019 from PHE Fingertips. These covariates were selected as variables where there were at least theoretical reasons to expect an influence on crisis care teams and mental health services. The selected covariates were: social deprivation (area deprivation score, adults in employment, adults in stable housing), which has been explore as a risk factor for admission and detention^17^ and has been found to be related to provision of crisis alternatives (e.g. crisis houses);^8^ psychiatric morbidity (psychosis prevalence), which has been proposed as a driver of admission and detention rates^18^ and may also influence the extent and nature of community crisis care provision; area-level demographic characteristics, as some groups in society (age, gender, ethnicity) may be at greater risk of admission, but face barriers to accessing community services, reducing the potential impact of community crisis services (e.g. people from minority ethnic groups are at higher risk of psychiatric admission and detention, but have lower use of community services than people from White ethnic groups);^19,20^ adults in contact with community services and percentage of patients with a crisis plan, as areas with well-resourced/efficient community services may have lower admission and detention rates.^21^ The latter two variables were selected as available indicators of the quality and reach of (non-crisis) community mental health services. The covariates were used as putative confounders in exploring relationships between crisis system characteristics and the outcome variables (hospital admission and detention rates).

### Statistical analysis

Descriptive statistics were used to summarise the service models and system characteristics, outcomes and covariates/confounders (mean, s.d., minimum and maximum). When there was evidence that the distribution of the data could be skewed, the median and the interquartile range replaced the mean and s.d. Furthermore, we conducted two separate analyses to address our two objectives.

#### Latent class analysis: typologies of mental health local crisis care systems

We used latent class analysis (LCA) to explore whether, based on our binary national survey data (presence/absence of service models and systems characteristics), a typology of crisis care systems at the CCG level could be derived. LCA is a structural equation modelling method that assumes that a set of observed binary variables can be modelled as a function of a several discrete unobserved (or latent) categories (or classes). More specifically, the modelling assumes that each binary outcome is ‘present’ once a threshold on an underlying continuous scale is exceeded and that given the class membership outcomes are independent. For a given number of classes, the parameters of this model can be estimated by maximum likelihood, and for each CCG, posterior probabilities of class memberships can be derived and used to classify CCGs *post hoc*. However, before any classification can take place, the number of classes needs to be estimated and, most importantly in the context of typologies, it needs to be demonstrated that there exists more than one class. To this end, we utilised Akaike's information criteria (AIC = 2*k* – 2ln(*L*), where *k* is the number of model parameters and *L* is the maximised value of the likelihood function; a model with lower AIC provided better fit) and Bayesian information criteria (BIC = *k*ln(*n*) – 2ln(*L*), where *k* is the number of parameters, *n* is the number of observations and *L* is the maximised value of the likelihood function; again, a model with lower BIC provides better fit) to compare the fit of two latent class models with different numbers of classes. We compared sequentially more complex models. Starting with a single class (no typology), we compared the one-class model with the two-class model, and so on.

#### Mixed-effects negative binomial models: characteristics of the crisis care systems and mental health hospital admissions and detentions

Mixed-effects negative binomial models were fitted to study the association between service models and system characteristics with mental health hospital admissions and detentions at CCG level. The two main outcomes (mental health admissions and detentions) were counts from April 2018 to March 2019. Given the initial variables were in the form of rates per 100 000 person-years, counts of mental health admissions and detentions were calculated by multiplying the rates by the CCG resident population and then dividing by 100 000. As the CCGs were clustered within 54 NHS Trusts, a random effect for NHS Trust (random intercept) was included to account for within-trust correlation between CCG measures. In addition, the population size by CCG in 2018 was log-transformed and included as an offset variable in the models. Since the population size in 2018 was not available in four CCGs, the rate per 100 000 person-years was used as the count, and the population size was set at 100 000 for these four CCGs. The service models of triage wards and crisis family placements were excluded from the analysis because of their small numbers within crisis care systems.

First, mixed-effects negative binomial models were fitted to explore the association between each theoretically derived variable (access, choice and integration) and mental health admissions and detentions, unadjusted and adjusted for covariates (employment, accommodation, prevalence of psychosis, patients with crisis care plans, area deprivation score, contact with mental health services, Black and minority ethnic groups, gender and age). Second, several mixed-effects negative binomial models were conducted to analyse the relationship between each service model and system characteristics, and mental health admissions and detections, unadjusted and adjusted for covariates. The mixed-effects negative binomial models have the following algebraic form:
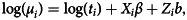
where log(*t*_*i*_) is the offset for the population size in each CCG, ***β*** is the vector for the regression coefficients for the fixed effects ***X***_*i*_ (characteristics of crisis care systems and covariates) and ***b*** is the vector for the regression coefficients for the random effects ***Z***_*i*_ (including the random intercept for the NHS Trust).

Statistical significance level was set at *P* < 0.05. All analyses were performed in Stata version 17 for Windows.

### Ethics

The 2019 survey,^2^ commissioned by national policy makers to understand current service provision, met national guidelines for a service evaluation and therefore did not require review from an ethics committee.^22^ Survey respondents were sent an information sheet and invitation email, and consented to take part by participating in the survey. We consulted Noclor, the research support service overseeing research governance for several NHS Trusts in North London, to confirm it was appropriate to treat this study as a service evaluation. This paper from our research team reports additional analyses using survey data and publicly available data from NHS Fingertips, for which no additional ethical review is required.

## Results

The summary of the frequency of service models and systems characteristics are presented in Table 2. Those service models most frequently found in CCGs were police street triage (65%), crisis telephone line (63%) and crisis houses (52%). Common system characteristics were integrated staffing (74%) and voluntary sector involvement (66%). There were some missing data for some variables within CCGs, ranging from 16 to 39 responses.

**Table 2:** Summary of service model and system characteristics

Variable	Number of CCGs (*N* = 195)	CCGs where present, *n* (%)
Service models		
24/7 crisis resolution team	172	77 (44)
Crisis assessment team	179	65 (36)
Crisis telephone line	177	112 (63)
Psychiatric decision unit	175	33 (18)
Triage ward	169	6 (3)
Crisis cafe	174	54 (31)
Police street triage	174	114 (65)
Ambulance street triage	175	35 (20)
Crisis house	173	90 (52)
Acute day units	175	21 (12)
Crisis family placement	169	2 (1)
System characteristics		
Integrated management	174	32 (18)
Integrated staffing	178	133 (74)
Voluntary sector	153	102 (66)
Peer-led	156	24 (15)

CCG, clinical commissioning group.

Table 3 shows the summary statistics for the outcome variables (mental health admissions and detentions) and for the covariates. To provide more explanatory information, median rates of admissions and detentions per 100 000 population are included in the table. In the analyses, counts (adjusted by population in CCGs) are used.

**Table 3:** Summary of outcomes and covariates

Variable	*n*	Median	IQR	Minimum	Maximum
Outcomes (CCG level)					
Mental health hospital admissions per 100 000 population (2018–2019)	190	666.18	557.99	59.64	800.07
Persons detained under MHA per 100 000 population (2018–2019)	191	841.99	751.83	29.82	1135.52

	*n*	Mean	s.d.	Minimum	Maximum
Covariates					
Percentage of CPA adults in employment (2018–2019)	180	8.96	3.91	1.99	23.43
Percentage of CPA adults in stable accommodation (2018–2019)	192	59.17	19.31	4.35	88.35
Mental health (psychosis) prevalence (%), all ages (2018–2019)	195	0.95	0.19	0.62	1.59
Percentage of patients with crisis plans (2018–2019)	192	12.42	19.31	0.079	72.13
Area deprivation score (IMD 2019)	189	21.92	8.03	7.18	52.13
People in contact with adult mental health services per 100 000 persons (2018–2019)^a^	195	2222.15	912.16	880.14	5488.58
Percentage of population from Black and minority ethnic groups (2011)	195	13.80	15.50	1.22	72.15
Percentage of population who are male (2017)	195	49.38	0.75	47.62	52.86
Median age of population, years (2017)	195	40.41	4.73	27	51

IQR, interquartile range; CCG, clinical commissioning group; MHA, Mental Health Act; CPA, care programme approach; IMD, Index of Multiple Deprivation.

a. Median and interquartile range.

### What types of mental health crisis care system are there at the CCG level?

The first step in the analysis involved trying to obtain a typology of mental health catchment area crisis systems. After conducting several LCA prespecifying that the analysis should derive two and three different classes, the AIC and BIC suggested that there was no evidence that those models provide a better fit than those with only one class. The models were gradually compared to check how they fitted (i.e. one class versus two classes, two classes versus three classes). The majority of the models did not converge, and the models that converged showed large variations within categories. Therefore, the resulting categories did not have a meaningful clinical interpretation in terms of potential typologies for local crisis care systems. These results are presented in Supplementary Appendix 2.

### What are the characteristics of crisis care systems associated with in-patient admissions and detentions?

Further planned analysis investigated associations between (a) crisis service models available in catchment areas and service use and (b) variables characterising the system as a whole (access, choice and integration) and service use. The adjusted analyses for crisis service models suggested that the presence of a crisis telephone line may be associated with a 11.6% lower level of mental health admissions (incidence rate ratio (IRR) 0.884, 95% CI 0.809–0.965), whereas the presence of a crisis cafe is associated with a 7.8% lower level of in mental health admissions. By contrast, having a crisis assessment team, separate from the home treatment team, was associated with a 12.8% higher rate in mental health admissions (IRR = 1.128, 95% CI 1.035–1.231). The presence of a crisis telephone line was also associated with a 15.3% lower rate of mental health detentions (IRR = 0.847, 95% CI 0.742–0.967). Other service models and all of the system characteristics we explored were not associated with admissions and detentions. Our theoretically derived variables (access, choice and integration) did not show any association with admissions or detentions (Table 4).

**Table 4:** Mixed-effects negative binomial models for crisis care characteristics and mental health admissions and detentions

Outcome	Crisis care characteristic	Unadjusted			Adjusted		
		*n*	IRR	95% CI	*n*	IRR	95% CI
Mental health admission							
	Theoretically derived						
Admission rate	Access	170	1.018	0.991–1.046	148	1.002	0.982–1.021
	Choice	155	0.973	0.942–1.005	135	0.987	0.965–1.011
	Integration	147	0.981	0.939–1.024	129	0.985	0.955–1.017
	Service models						
Admission rate	CRT 24/7	170	1.080	0.961–1.213	148	0.957	0.874–1.048
	Crisis assessment team	177	1.089	0.969–1.223	155	1.128**	1.035–1.231
	Crisis telephone line	175	0.916	0.812–1.033	155	0.884**	0.809–0.965
	Psychiatric decision unit	173	0.781*	0.646–0.946	151	0.871	0.742–1.022
	Crisis cafe	172	0.931	0.827–1.048	150	0.922*	0.849–0.999
	Police street triage	172	0.953	0.833–1.091	150	0.922	0.835–1.019
	Ambulance street triage	173	0.759**	0.629–0.909	151	0.857	0.734–1.002
	Crisis house	171	0.947	0.847–1.061	149	0.977	0.903–1.057
	Acute day units	173	0.803*	0.678–0.950	151	1.008	0.873–1.164
	System characteristics						
Admission rate	Integrated management	172	1.038	0.899–1.198	150	1.037	0.935–1.150
	Integrated staffing	176	1.001	0.893–1.123	154	1.033	0.954–1.119
	Voluntary sector	151	0.964	0.848–1.099	131	0.973	0.898–1.055
	Peer-led	154	0.927	0.788–1.090	134	0.949	0.841–1.071
Mental health detentions							
	Theoretically derived						
Detention rate	Access	171	0.995	0.958–1.033	148	0.999	0.969–1.029
	Choice	156	0.979	0.937–1.023	135	0.988	0.956–1.021
	Integration	148	1.010	0.951–1.073	129	1.029	0.978–1.084
	Service models						
Detention rate	CRT 24/7	171	1.094	0.930–1.289	148	1.095	0.949–1.263
	Crisis assessment team	178	0.927	0.790–1.087	155	0.948	0.834–1.078
	Crisis telephone line	176	0.968	0.824–1.038	155	0.847*	0.742–0.967
	Psychiatric decision unit	174	1.019	0.811–1.281	151	1.145	0.938–1.396
	Crisis cafe	173	1.018	0.866–1.196	150	1.025	0.908–1.157
	Police street triage	173	1.199*	1.012–1.419	150	1.132	0.988–1.297
	Ambulance street triage	174	1.019	0.816–1.274	151	1.159	0.959–1.400
	Crisis house	172	0.948	0.815–1.102	149	0.937	0.836–1.052
	Acute day units	174	0.905	0.723–1.133	151	0.985	0.813–1.194
	System characteristics						
Detention rate	Integrated management	173	1.003	0.824–1.221	150	0.989	0.846–1.157
	Integrated staffing	177	0.959	0.817–1.127	154	0.988	0.873–1.118
	Voluntary sector	152	0.954	0.795–1.146	131	0.973	0.853–1.109
	Peer-led	155	0.906	0.728–1.127	134	0.944	0.794–1.121

National Health Service Trust included as a random effect (random intercept). Adjusted: potential confounding variables (employment, stable accommodation, prevalence of psychosis, proportion of patients with crisis care plans, area deprivation score, proportion of adults in contact with mental health services, proportion of population from Black and minority ethnic groups, percentage of population who are male, median age of population). IRR, incidence rate ratio; CRT, crisis resolution team.

*P < 0.05, **P < 0.01.

## Discussion

The study provides exploratory findings on the association of several mental health crisis service models with mental health admissions and detentions in England. The presence of certain models or characteristics seemed to be related to decreasing fewer admissions (e.g. crisis cafe, crisis telephone line) and detentions (e.g. crisis telephone line), whereas only having a separate crisis assessment team was apparently associated with an higher level of mental health admissions. The variables capturing the concepts of access, integration and choice were not associated with mental health admissions or detentions. Furthermore, the analyses exploring potential categories of crisis care systems did not yield any valid typology, which suggests that there may not be an established, coordinated pattern regarding how mental health crisis care systems are organised nationally.

Although a limited number of crisis service models^14^ and community service models^23^ have international evidence of effectiveness in reducing hospital admissions and cost-effectiveness, to the best of our knowledge, this is the first study that analyses the components of crisis care systems to explore whether there are the typologies of such systems, and to explore associations of system characteristics with mental health admissions and detentions. The findings from the adjusted models indicate that the presence of crisis telephone lines, such as a single point of access providing telephone triage, is associated with a lower rate in both admissions and detentions. According to a Care Quality Commission report,^10^ individuals experiencing a mental health crisis may benefit from crisis telephone services. However, to ensure effective delivery, it is essential to provide a service that is sensitive to individuals and accessible out of hours.^10^ Similarly, the results of the adjusted analyses indicate that the presence of a crisis cafe was associated with reduced mental health admissions, but not rates of detention. Crisis cafes are alternative services to hospital emergency departments, usually provided by the voluntary sector, where individuals experiencing a crisis may obtain support and signposting to other services.^2^ As this is a cross-sectional study, the causal relationships here are unclear. However, such a finding would be in keeping with findings that assessment in emergency departments is disproportionately likely to lead to admission,^24^ suggesting that providing a potentially more acceptable and accessible alternative to attending emergency departments and diverting people from this setting may be productive in reducing admissions. This finding suggests that access to a crisis cafe may help avoid admission. However, further investigations of a longitudinal nature are needed to confirm this possibility.

Initial local evaluations of early-adopter crisis assessment teams were promising, suggesting that teams focused only on crisis assessment and not on home treatment might prevent admissions more effectively than combined crisis assessment and home treatment teams.^25^ Nevertheless, we found that nationally, the presence of a stand-alone crisis assessment team in the local crisis system was associated with a higher rate of mental health admissions. Our research cannot elucidate the reasons for this cross-sectional finding. A possible explanation is that separating out initial crisis assessment and crisis home treatment into separate teams, and removing the ‘gatekeeping’ of hospital admissions from the service that provides crisis home treatment, may lead to discontinuities in care and more conservative risk management. CRTs, which provide both crisis assessment and home treatment, could be best placed to limit the access to hospital admissions where they are avoidable.

Triage through a crisis assessment team could, in some contexts, act as a barrier to prompt access to CRT support, which might also lead to an increase in admissions. Previous research has also suggested that longer opening hours^26^ and better access to CRTs^1^ were associated with lower admissions. However, our results did not show any significant association between access, or having a 24-h CRT, and lower rates of mental health admissions. Furthermore, other theoretically derived variables, such as integration and choice, did not show any association with either admissions or detentions. Likewise, other service models and system characteristics did not show any association with admissions and detentions.

The variables capturing the concepts of access, integration and choice were not associated with either outcome. This was counter to our expectations, as the variables were developed by a stakeholder group to capture aspects of crisis care system functioning reflecting good practice. Lack of association might reflect imprecision in data collection (one informant often provided information on a whole system), or lack of granularity in recording variables intended to capture the functioning of a whole system.

### Limitations

First, the survey data was self-reported, which could have led to some inaccuracies. PHE Fingertips data is likewise based on locally submitted data, and therefore some biases may be introduced if data were not consistently collected. The three theoretically derived variables (choice, integration and access) were not validated or checked for internal consistency, which may undermine their reliability and validity. Our study only explored cross-sectional relationships between variables and one period of time. The mixed-effect negative binomial regression models were fitted to test association rather than proving causation. In this regard, we were able to adjust for some potentially important covariates, but we cannot ensure that other covariates not included in the analysis may influence the results. Therefore, we could not discard the possibility that that unadjusted covariates or interactions between characteristics of the crisis care systems may influence the results, and that ecological fallacy may occur. Also, it was not feasible to use some originally proposed outcome variables (e.g. emergency department attendance), given the poor quality of the data (based on advice from NHS England). In addition, the analyses were not adjusted for multiple testing, which may increase the risk that some significant differences were because of chance. Some corrections could have been applied (e.g. Bonferroni correction). However, the main objective of the study was to explore the relationship between crisis care system characteristics and admissions and detentions for future investigations, rather than try to evaluate any impact on the association between variables. Another limitation is that data were collected before the COVID-19 outbreak, and many crisis care systems have been reorganised since then. Local commissioning areas in England have also been reorganised since 2019, with CCGs replaced by larger area-level integrated care boards.^27^

### Implications for policy, practice and research

Given that our findings were exploratory, the conclusions are preliminary. Our study does not provide evidence to support any policy recommendation for creating new crisis assessment teams, but splitting the crisis assessment and crisis home treatment functions into different teams instead. Conversely, the positive results for a crisis telephone line suggest that 24-h access to crisis support may be important. Our study also provides preliminary evidence that introducing crisis cafes into local crisis care systems could potentially help to reduce admission rates. Overall, the analyses yielded few associations between what service types are included within a local crisis care system and local admissions and detentions. Investing time and money in setting up innovative new crisis services has an opportunity cost. Rather than invest in a proliferation of new crisis service models, local commissioners and service planners may be better advised to focus energies and resources on improving the quality of care in their current local crisis system, which may also help to reduce in-patient admissions.^28^ Our finding that no valid typology of crisis care systems could be ascertained may reflect that mental health crisis services are developed and commissioned piecemeal over time, rather than as clearly conceptualised, systemic responses to local needs. Further research is required to identify effective crisis care systems configurations and how they may best be implemented according to local needs. In this regard, lived experience researchers provided their independent views about the results of the study, highlighting those areas of research in crisis care systems that may be essential to cover in future investigations.

Further research is needed to clarify these exploratory findings. More robust methodological approaches, such as longitudinal designs (i.e. a time series of controlled studies), may help to reveal the association between crisis care system characteristics and mental health admissions and detentions. Additionally, this research could explore the specification of models for innovative crisis care services and development and evaluation of quality improvement programmes, such as the evaluation of crisis cafes. Other approaches investigating psychiatric admissions and detentions, and the implementation of certain models, may also help to clarify the direction of potential associations.

### Lived experience commentary

#### Tamar Jeynes and Lizzie Mitchell

We welcome this exploration into different models of crisis care – a much needed investigation. Preventing admissions is a crucial part of crisis care, but it should not be the sole focus. Measuring only admission rates does not capture the other benefits crisis care should bring, such as having human connection, helping people to manage extreme levels of distress, preventing harm to themselves and others, or dying by suicide. We understand that this is hard to measure, but the true effect of crisis services cannot just be judged by admissions – it needs to be what patients feel helps them at their time of need.

Statistical methods provide some insight into what may work. However, the variance of the services cannot be captured. As an example, crisis houses and cafes can be NHS, third sector or peer-run, each using different approaches that may, at times, contradict the approach of another using the same name.

The finding that crisis telephone lines reduce admissions is of interest because of the wide variation of efficacy between different areas. We wondered if exceptional areas masked the failings of other areas.

Mental health development begins in childhood, yet children and young people's services are not included in this research. To change the way services are structured, we need to change this. Children and young people's crisis services are often structured very differently to adult services. These should be included so that we can see crisis care across a continuum and assess effective structure of services.

The data for this study was collected in 2019, before the COVID-19 pandemic. The pandemic has led to increased funding for crisis healthcare, including research co-produced with lived experience researchers, which readers could compare for changes or corroborating findings from other research. Further exploration into such research should be a priority to ensure services are improved for people at the time when they need them the most.

The findings themselves are of interest beyond the scientific community, and we welcome the National Institute for Health and Care Research (NIHR) Mental Health Policy Research Unit's plans to make plain English summary accessible to lay people.

## Supporting information

Rojas-García et al. supplementary material Rojas-García et al. supplementary material

## Data Availability

De-identified data (anonymised) are available from the corresponding author, A.R.-G., on request.
