# Investigating the association between characteristics of local crisis care systems and service use in an English national survey – CORRIGENDUM

**DOI:** 10.1192/bjo.2023.631

**Published:** 2023-12-06

**Authors:** Antonio Rojas-García, Christian Dalton-Locke, Luke Sheridan Rains, Ceri Dare, Cedric Ginestet, Una Foye, Kathleen Kelly, Sabine Landau, Chris Lynch, Paul McCrone, Shilpa Nairi, Karen Newbigging, Patrick Nyikavaranda, David Osborn, Karen Persaud, Nick Sevdalis, Martin Stefan, Ruth Stuart, Alan Simpson, Sonia Johnson, Brynmor Lloyd-Evans

**Keywords:** Mental health services, crisis care models and systems, psychiatric admissions, psychiatric detentions, England, corrigendum

This article was originally published with an error in the affiliation of author David Osborn. He is in fact affiliated with the Division of Psychiatry, University College London, UK, as well as the Camden and Islington NHS Foundation Trust, London, UK. This has now been corrected and this corrigendum published.
