# Supplementary material for: Investigating the association between characteristics of local crisis care systems and service use in an English national survey
Source: BJPsych Open. 2023 Nov 3;9(6):e209. doi: 10.1192/bjo.2023.595 (PMC10753954; doi:10.1192/bjo.2023.595)
Supplement: Rojas-García et al. supplementary material [file S2056472423005951sup001.docx]

**Appendix 1. Access, Choice, Integration (theoretically derived variables)**

**Scoring rules:**

Note: Each variable is first scored at the CRT-level before being transformed to the CCG-level according to the following rules.

1. When coded at the CRT-level, variables were scored as missing where insufficient data were available to establish whether a score of 0, 1 or 2 was achieved
2. All variables score zero if they do not score one or two and are not missing
3. The Domain total = missing if any of the variables scoring on to the domain are missing
4. At the CCG-level score each variable if data are available for at least 50% of CRTs serving this CCG – otherwise count as missing
5. Each criterion as met if at least 50% of CRTs which provided data for this CCG meet the criterion

| **Table 1: Domain 1: Access (Total score 0-8)** | | | |
| --- | --- | --- | --- |
| **Variable** | **Scoring** | **Rationale: why is this important to people in crisis?** | **Score at CCG-level (N=195)** |
| 24/7 access | 2: There is a Crisis Assessment Team which is open 24/7 OR There is a CRT or Crisis line which offers telephone support 24/7 and  the CRT sees people in person 24/7 (including home visits or seeing patients on NHS premises)  1: There is a crisis line or CRT offering telephone assessment and support which runs 24/7 but no Crisis assessment Service or CRT which can see people in person 24/7 | Services are running whatever time I need to access crisis support | 0 = 13 (7%)  1 = 34 (19%)  2 = 129 (73%)  Missing = 19 (10%) |
| Self-referral | 2: People known and not known to services can self-refer to the Crisis Assessment Team or CRT  1. People known to services can self-refer to the Crisis Assessment Team or CRT | I can access crisis services directly when I need to | 0 = 58 (33%)  1 = 33 (19%)  2 = 83 (48%)  Missing = 21 (11%) |
| Referral from other agencies | One point each for:  1: The Crisis Assessment Team or CRT accepts referrals from emergency services (police and/or ambulance)  1: The Crisis Assessment Team or CRT accepts referrals from GPs | Other professionals trying to help me can access crisis services directly when they need to | 0 = 28 (16%)  1 = 29 (17%)  2 = 117 (67%)  Missing = 21 (11%) |
| 24/7 CRT response | 2: The CRT can see current service users at home 24/7  1: The CRT can see current service users on NHS premises 24/7 | The Crisis Team who are supporting me can see me conveniently whatever time I need help | 0 = 76 (39%)  1 = 29 (15%)  2 = 84 (43%)  Missing = 6 (3%) |

| **Table 2: Domain 2: Choice (Total score 0-6)** | | | |
| --- | --- | --- | --- |
| **Variable** | **Scoring** | **Rationale: why is this important to people in crisis?** | **Score at CCG-level (N=195)** |
| Alternative places to seek help in a crisis  (Crisis cafes) | 2: There is an immediately accessible place other than A&E where people can go in a mental health crisis (e.g. a crisis café with drop-in or self-referral)  1: There is an alternative to A&E e.g. a crisis café, but access is via referral from professionals | I don’t have to go to A&E if I feel unsafe at home: there is an alternative | 0 = 119 (69%)  1 = 10 (6%)  2 = 44 (25%)  Missing = 22 (11%) |
| Alternatives to treatment at home in a crisis | 2: There are at least two different crisis alternatives to home treatment or hospital (crisis house, Acute day Unit, family placement scheme)  1: There is at least one crisis alternative to hospital or home treatment | There are other options not just home treatment, if I need another environment to support my recovery | 0 = 76 (45%)  1 = 78 (46%)  2 = 14 (8%)  Missing = 27 (14%) |
| Availability of alternatives to treatment at home in a crisis  (ADUs, crisis houses or crisis family placements) | One point each for:  1: The CRT reports at least one crisis alternative usually has spaces available when required  1: Admission to at least one crisis alternative can usually be arranged within 24 hours | And other options in addition to home treatment are usually available | 0 = 76 (47%)  1 = 9 (6%)2 = 76 (47%)  Missing = 34 (17%) |
| *Types of support | One point each for:  1: The local crisis system includes at least one non-NHS provided service (from survey responses or Karen Newbigging project list)  1: At least one crisis service is service user-led | If I have not found typical NHS healthcare helpful, other types of support are offered | - |
| *Dropped due to high missing rate | | | |

| **Table 3: Domain 3: Integration (Total score 0-6)** | | | |
| --- | --- | --- | --- |
| **Variable** | **Scoring** | **Rationale: why is this important to people in crisis?** | **Score at CCG-level (N=195)** |
| Within mental health crisis services | 2: CRHTT care can be accessed via a single assessment (e.g. the CRHTT accepts self-referrals from all; or the Crisis Assessment Team accepts self-referrals from all and can arrange CRHTT care without reassessment)  1: There are two steps to CRHTT care in some or all circumstances (e.g. the CRHTT accepts referrals directly from all health professionals but not self-referrals from all; OR referrals are via the Crisis Assessment Team, which accepts self-referrals, but the CRHTT re-assesses referrals from the Crisis Assessment Team before accepting people for home treatment) | I can get crisis support without having to tell my story repeatedly to different professionals | 0 = 79 (45%)  1 = 24 (14%)  2 = 74 (43%)  Missing = 18 (9%) |
| With acute wards | 2: The CRHTT (or Crisis Assessment Team) report that they seek to gatekeep at least 95% of people in person before admission to an acute inpatient ward  1: The CRHTT report that they seek to gatekeep at least 50% of people in person. | I will not be admitted to hospital unless home treatment and crisis alternatives have been fully considered | 0 = 18 (11%)  1 = 55 (33%)  2 = 92 (56%)  Missing = 30 (15%) |
| With emergency services | One point each for:  1: There is a police street triage team  1: There is an ambulance street triage team | If I come into contact with the police or ambulance staff during a crisis, they can access help for me quickly, in the least restrictive way. | 0 = 58 (36%)  1 = 81 (50%)  2 = 24 (15%)  Missing = 32 (16%) |

**Appendix 2. Summary of Latent Class Analysis for Crisis Care Teams Project**

**CRT 24/7; Crisis assessment; Crisis café; Crisis house (Missing= 29)**

1-Class

Number of observations = 179

| **Latent class marginal probabilities** | | | | | |
| --- | --- | --- | --- | --- | --- |
|  | Class | Margin | SE | [95% conf. interval] | |
|  | 1 | 1 | - | - | - |
|  |  |  |  |  |  |

| **Latent class marginal means** | | | | | |
| --- | --- | --- | --- | --- | --- |
| Class |  | Margin | SE | [95% conf. interval] | |
| 1 |  |  |  |  |  |
|  | CRT 24/7 | .4476744 | .0379153 | .3750497 | .5226022 |
|  | Crisis assessment | .3631285 | .0359443 | .2959964 | .4360555 |
|  | Crisis café | .3103448 | .0350723 | .246071 | .382881 |
|  | Crisis house | .5202312 | .0379832 | .4458873 | .5936898 |
|  |  |  |  |  |  |

| **Fit statistic** | **Value** |  |
| --- | --- | --- |
| Information criteria |  |  |
| AIC Akaike's information | 934.205 |  |
| BIC Bayesian information | 946.954 |  |

2-Classes

Number of observations = 179 / *Convergence not achieved*

| **Latent class marginal probabilities** | | | | | |
| --- | --- | --- | --- | --- | --- |
|  | Class | Margin | SE | [95% conf. interval] | |
|  | 1 | .5554708 | .0378189 | .4806624 | .6278466 |
|  | 2 | .4445292 | .0378189 | .3721534 | .5193376 |

| **Latent class marginal means** | | | | | |
| --- | --- | --- | --- | --- | --- |
| Class |  | Margin | SE | [95% conf. interval] | |
| 1 |  |  |  |  |  |
|  | CRT 24/7 | 1.52e-08 | . | . | . |
|  | Crisis assessment | .4452592 | .0502202 | .3501591 | .5445444 |
|  | Crisis café | .2402155 | .0437242 | .1650608 | .3358259 |
|  | Crisis house | .4719129 | .0508186 | .3746924 | .5713117 |
| 2 |  |  |  |  |  |
|  | CRT 24/7 | .9999997 | .0003351 | 0 | 1 |
|  | Crisis assessment | .2604992 | .0498189 | .1750507 | .3690011 |
|  | Crisis café | .3961222 | .0554908 | .2939423 | .5082531 |
|  | Crisis house | .5825733 | .0573173 | .4678912 | .6889691 |

| **Fit statistic** | **Value** |  |
| --- | --- | --- |
| Information criteria |  |  |
| AIC Akaike's information | 929.195 |  |
| BIC Bayesian information | 954.695 |  |

3-Classes

Number of observations = 179 / *Convergence not achieved*

| **Latent class marginal probabilities** | | | | | |
| --- | --- | --- | --- | --- | --- |
|  |  | Margin | SE | [95% conf. interval] | |
|  | Class |  |  |  |  |
|  | 1 | .5539351 | .0378343 | .4791309 | .6263736 |
|  | 2 | .1701065 | .0285199 | .12123 | .2334537 |
|  | 3 | .2759584 | .0337991 | .2148143 | .346819 |

| **Latent class marginal means** | | | | | |
| --- | --- | --- | --- | --- | --- |
|  |  | Margin | SE | [95% conf. interval] | |
| 1 |  |  |  |  |  |
|  | CRT 24/7 | .0345358 | .6436248 | 1.33e-18 | 1 |
|  | Crisis assessment | .6871114 | .11209 | .441463 | .8591835 |
|  | Crisis café | .3743772 | .0860893 | .225502 | .5515459 |
|  | Crisis house | .4434891 | .0711958 | .3116535 | .5837954 |
| 2 |  |  |  |  |  |
|  | CRT 24/7 | .3403855 | .34927 | .0238808 | .9158582 |
|  | Crisis assessment | 1.32e-06 | .0010443 | 0 | 1 |
|  | Crisis café | 5.60e-08 | .0001567 | 0 | 1 |
|  | Crisis house | .5847946 | .1455175 | .3031945 | .820112 |
| 3 |  |  |  |  |  |
|  | CRT 24/7 | .9999999 | .0004119 | 0 | 1 |
|  | Crisis assessment | .3229495 | .1875192 | .0815751 | .7192281 |
|  | Crisis café | .5172426 | .3316252 | .0735093 | .9353533 |
|  | Crisis house | .5505283 | .0879703 | .3789553 | .7108664 |

| **Fit statistic** | **Value** |  |
| --- | --- | --- |
| Information criteria |  |  |
| AIC Akaike's information | 932.166 |  |
| BIC Bayesian information | 976.789 |  |

**CRT 24/7; Crisis assessment; Crisis telephone; Crisis café; Crisis house (Missing= 31)**

1-Classes

Number of observations = 179

| **Latent class marginal probabilities** | | | | | |
| --- | --- | --- | --- | --- | --- |
|  |  | Margin | SE | [95% conf. interval] | |
|  | Class |  |  |  |  |
|  | 1 | 1 | - | - | - |
|  |  |  |  |  |  |

| **Latent class marginal means** | | | | | |
| --- | --- | --- | --- | --- | --- |
|  |  | Margin | SE | [95% conf. interval] | |
| 1 |  |  |  |  |  |
|  | CRT 24/7 | .4476744 | .0379153 | .3750497 | .5226022 |
|  | Crisis assessment | .3631285 | .0359443 | .2959964 | .4360555 |
|  | Crisis telephone | .6327684 | .0362331 | .5593441 | .700509 |
|  | Crisis café | .3103448 | .0350723 | .246071 | .382881 |
|  | Crisis house | .5202312 | .0379832 | .4458873 | .5936898 |
|  |  |  |  |  |  |

| **Fit statistic** | **Value** |  |
| --- | --- | --- |
| Information criteria |  |  |
| AIC Akaike's information | 1168.947 |  |
| BIC Bayesian information | 1184.884 |  |

2-Classes

Number of observations = 179

| **Latent class marginal probabilities** | | | | | |
| --- | --- | --- | --- | --- | --- |
|  |  | Margin | SE | [95% conf. interval] | |
|  | Class |  |  |  |  |
|  | 1 | .5555128 | .0378222 | .4806969 | .6278938 |
|  | 2 | .4444872 | .0378222 | .3721062 | .5193031 |

| **Latent class marginal means** | | | | | |
| --- | --- | --- | --- | --- | --- |
|  |  | Margin | SE | [95% conf. interval] | |
| 1 |  |  |  |  |  |
|  | CRT 24/7 | 2.30e-08 | .0001013 | 0 | 1 |
|  | Crisis assessment | .4452945 | .0502243 | .3501856 | .5445864 |
|  | Crisis telephone | .6414416 | .0490236 | .540873 | .7309398 |
|  | Crisis café | .2403075 | .0437385 | .1651261 | .3359449 |
|  | Crisis house | .4720504 | .0508291 | .3748052 | .571464 |
| 2 |  |  |  |  |  |
|  | CRT 24/7 | .9999987 | .0006617 | 0 | 1 |
|  | Crisis assessment | .2604408 | .049813 | .1750051 | .3689335 |
|  | Crisis telephone | .622146 | .0549343 | .5101627 | .7224561 |
|  | Crisis café | .395982 | .0554816 | .2938248 | .5081015 |
|  | Crisis house | .5823834 | .0573183 | .4677095 | .6887909 |

| **Fit statistic** | **Value** |  |
| --- | --- | --- |
| Information criteria |  |  |
| AIC Akaike's information | 1167.871 |  |
| BIC Bayesian information | 1202.932 |  |

3-Classes

Number of observations = 179

| **Latent class marginal probabilities** | | | | | |
| --- | --- | --- | --- | --- | --- |
|  |  | Margin | SE | [95% conf. interval] | |
|  | Class |  |  |  |  |
|  | 1 | .3727462 | .274018 | .0563856 | .8552762 |
|  | 2 | .2958648 | .1292037 | .1107975 | .5862502 |
|  | 3 | .331389 | .3740782 | .0177942 | .9313176 |

| **Latent class marginal means** | | | | | |
| --- | --- | --- | --- | --- | --- |
|  |  | Margin | SE | [95% conf. interval] | |
| 1 |  |  |  |  |  |
|  | CRT 24/7 | 4.32e-08 | .0002247 | 0 | 1 |
|  | Crisis assessment | .4463932 | .0503089 | .3510892 | .5458077 |
|  | Crisis telephone | .6443354 | .0490391 | .5435945 | .733732 |
|  | Crisis café | .2410937 | .0438621 | .1656816 | .3369673 |
|  | Crisis house | .4733808 | .0509116 | .3759318 | .5729008 |
| 2 |  |  |  |  |  |
|  | CRT 24/7 | .9999997 | .0004039 | 0 | 1 |
|  | Crisis assessment | .2745558 | .0819669 | .1445233 | .4588331 |
|  | Crisis telephone | 2.13e-07 | .0004121 | 0 | 1 |
|  | Crisis café | .1970703 | .0723206 | .091081 | .3754501 |
|  | Crisis house | .4754652 | .0931448 | .303598 | .6533475 |
| 3 |  |  |  |  |  |
|  | CRT 24/7 | .9999997 | .0004865 | 0 | 1 |
|  | Crisis assessment | .25062 | .0623594 | .1485417 | .3906618 |
|  | Crisis telephone | 1 | . | . | . |
|  | Crisis café | .519123 | .0721081 | .3799841 | .6553578 |
|  | Crisis house | .6468111 | .0706263 | .4997778 | .7704744 |

| **Fit statistic** | **Value** |  |
| --- | --- | --- |
| Information criteria |  |  |
| AIC Akaike's information | 1167.358 |  |
| BIC Bayesian information | 1218.356 |  |

**CRT 24/7; Crisis assessment; Crisis tel; Crisis café; Crisis house; PDU (Missing= 32)**

1-Class

Number of observations = 179

| **Latent class marginal probabilities** | | | | | |
| --- | --- | --- | --- | --- | --- |
|  |  | Margin | SE | [95% conf. interval] | |
|  | Class |  |  |  |  |
|  | 1 | 1 | - | - | - |
|  |  |  |  |  |  |

| **Latent class marginal means** | | | | | |
| --- | --- | --- | --- | --- | --- |
| Class |  | Margin | SE | [95% conf. interval] | |
| 1 |  |  |  |  |  |
|  | CRT 24/7 | .4476744 | .0379153 | .3750497 | .5226022 |
|  | Crisis assessment | .3631285 | .0359443 | .2959964 | .4360555 |
|  | Crisis telephone | .6327684 | .0362331 | .5593441 | .700509 |
|  | Crisis café | .1885714 | .0295695 | .1372784 | .2534005 |
|  | Crisis house | .3103448 | .0350723 | .246071 | .382881 |
|  | PDU | .5202312 | .0379832 | .4458873 | .5936898 |

| **Fit statistic** | **Value** |  |
| --- | --- | --- |
| Information criteria |  |  |
| AIC Akaike's information | 1340.398 |  |
| BIC Bayesian information | 1359.522 |  |

2-Classes

Number of observations = 179 / *Convergence not achieved*

| **Latent class marginal probabilities** | | | | | |
| --- | --- | --- | --- | --- | --- |
|  |  | Margin | SE | [95% conf. interval] | |
|  | Class |  |  |  |  |
|  | 1 | .5539602 | .0378754 | .4790743 | .6264743 |
|  | 2 | .4460398 | .0378754 | .3735257 | .5209257 |

| **Latent class marginal means** | | | | | |
| --- | --- | --- | --- | --- | --- |
| Class |  | Margin | SE | [95% conf. interval] | |
| 1 |  |  |  |  |  |
|  | CRT 24/7 | 1.52e-08 | . | . | . |
|  | Crisis assessment | .4450777 | .050314 | .3498153 | .5445547 |
|  | Crisis telephone | .6421844 | .0490932 | .5414328 | .7317664 |
|  | Crisis café | .2703199 | .0453894 | .1909242 | .367726 |
|  | Crisis house | .2408195 | .0438239 | .1654805 | .3366226 |
|  | PDU | .47329 | .0509288 | .3758128 | .5728467 |
| 2 |  |  |  |  |  |
|  | CRT 24/7 | .9999991 | .0004338 | 0 | 1 |
|  | Crisis assessment | .2613521 | .0498498 | .1757999 | .3698549 |
|  | Crisis telephone | .6213097 | .0548982 | .5094482 | .7216025 |
|  | Crisis café | .0888246 | .0320703 | .0429136 | .1748786 |
|  | Crisis house | .3949067 | .0553963 | .2929488 | .5069093 |
|  | PDU | .5803924 | .0573031 | .4658569 | .686876 |

| **Fit statistic** | **Value** |  |
| --- | --- | --- |
| Information criteria |  |  |
| AIC Akaike's information | 1329.448 |  |
| BIC Bayesian information | 1367.697 |  |

3-Classes

Number of observations = 179

| **Latent class marginal probabilities** | | | | | |
| --- | --- | --- | --- | --- | --- |
|  |  | Margin | SE | [95% conf. interval] | |
|  | Class |  |  |  |  |
|  | 1 | .0534656 | .0180223 | .0273381 | .1019466 |
|  | 2 | .689049 | .0350847 | .6165044 | .7533606 |
|  | 3 | .2574854 | .0336828 | .1971218 | .3287646 |

| **Latent class marginal means** | | | | | |
| --- | --- | --- | --- | --- | --- |
| Class |  | Margin | SE | [95% conf. interval] | |
| 1 |  |  |  |  |  |
|  | CRT 24/7 | .0945528 | .0996841 | .0105463 | .5057092 |
|  | Crisis assessment | 1 | .0000974 | 0 | 1 |
|  | Crisis telephone | .3858344 | .1722557 | .1312906 | .7230983 |
|  | Crisis café | .9999999 | .0001548 | 0 | 1 |
|  | Crisis house | .9999997 | .0006067 | 0 | 1 |
|  | PDU | .931722 | .1185423 | .2613908 | .9981031 |
| 2 |  |  |  |  |  |
|  | CRT 24/7 | .3916551 | .0450861 | .3076256 | .4826377 |
|  | Crisis assessment | .3079593 | .041842 | .232459 | .3953505 |
|  | Crisis telephone | .5807252 | .0449524 | .4909757 | .6654336 |
|  | Crisis café | .1744066 | .0345922 | .1165434 | .2527786 |
|  | Crisis house | 4.89e-07 | .0002557 | 0 | 1 |
|  | PDU | .5215281 | .0457252 | .4321759 | .6095236 |
| 3 |  |  |  |  |  |
|  | CRT 24/7 | .6690852 | .072117 | .5164085 | .7928914 |
|  | Crisis assessment | .3785024 | .0737521 | .2477965 | .5296102 |
|  | Crisis telephone | .8210485 | .0573542 | .6810025 | .9079244 |
|  | Crisis café | .0593013 | .0411319 | .0146416 | .2110105 |
|  | Crisis house | .9999998 | .0004226 | 0 | 1 |
|  | PDU | .4365183 | .0753899 | .2981686 | .5855078 |

| **Fit statistic** | **Value** |  |
| --- | --- | --- |
| Information criteria |  |  |
| AIC Akaike's information | 1320.872 |  |
| BIC Bayesian information | 1384.620 |  |

**CRT 24/7; Crisis assessment; Crisis tel; Crisis café; Crisis house; PDU; Police st triage (Missing= 34)**

1-Class

Number of observations = 179

| **Latent class marginal probabilities** | | | | | |
| --- | --- | --- | --- | --- | --- |
|  |  | Margin | SE | [95% conf. interval] | |
|  | Class |  |  |  |  |
|  | 1 | 1 | - | - | - |
|  |  |  |  |  |  |

| **Latent class marginal means** | | | | | |
| --- | --- | --- | --- | --- | --- |
| Class |  | Margin | SE | [95% conf. interval] | |
| 1 |  |  |  |  |  |
|  | CRT 24/7 | .4476744 | .0379153 | .3750497 | .5226022 |
|  | Crisis assessment | .3631285 | .0359443 | .2959964 | .4360555 |
|  | Crisis telephone | .6327684 | .0362331 | .5593441 | .700509 |
|  | Crisis café | .1885714 | .0295695 | .1372784 | .2534005 |
|  | Crisis house | .3103448 | .0350723 | .246071 | .382881 |
|  | Police St Triage | .6551724 | .0360333 | .5815768 | .7220108 |
|  | PDU | .5202312 | .0379832 | .4458873 | .5936898 |
|  |  |  |  |  |  |

| **Fit statistic** | **Value** |  |
| --- | --- | --- |
| Information criteria |  |  |
| AIC Akaike's information | 1566.575 |  |
| BIC Bayesian information | 1588.887 |  |

2-Classes

Number of observations = 179

| **Latent class marginal probabilities** | | | | | |
| --- | --- | --- | --- | --- | --- |
|  |  | Margin | SE | [95% conf. interval] | |
|  | Class |  |  |  |  |
|  | 1 | .3231165 | .1144055 | .1462005 | .5709542 |
|  | 2 | .6768835 | .1144055 | .4290458 | .8537995 |

| **Latent class marginal means** | | | | | |
| --- | --- | --- | --- | --- | --- |
| Class |  | Margin | SE | [95% conf. interval] | |
| 1 |  |  |  |  |  |
|  | CRT 24/7 | .6226942 | .1206966 | .3761612 | .8187458 |
|  | Crisis assessment | .1798788 | .0819247 | .0687782 | .3944301 |
|  | Crisis telephone | .9597707 | .1147179 | .065915 | .999876 |
|  | Crisis café | .0262711 | .0468819 | .0007426 | .4948362 |
|  | Crisis house | .4810073 | .1201858 | .2651038 | .7042447 |
|  | Police St Triage | .4495749 | .0992657 | .271186 | .6419498 |
|  | PDU | .5310438 | .1111738 | .3206854 | .7309198 |
| 2 |  |  |  |  |  |
|  | CRT 24/7 | .3627671 | .0640025 | .2486131 | .4948189 |
|  | Crisis assessment | .4506044 | .0694685 | .3212094 | .5870447 |
|  | Crisis telephone | .4755803 | .0747058 | .335182 | .6199477 |
|  | Crisis café | .2655239 | .0549743 | .1722242 | .3858099 |
|  | Crisis house | .2287464 | .0494502 | .1461936 | .3393855 |
|  | Police St Triage | .7524104 | .0637696 | .6083981 | .8559973 |
|  | PDU | .5151263 | .061289 | .3964178 | .6321522 |

| **Fit statistic** | **Value** |  |
| --- | --- | --- |
| Information criteria |  |  |
| AIC Akaike's information | 1563.360 |  |
| BIC Bayesian information | 1611.171 |  |

3-Classes

Number of observations = 179 / *Convergence not achieved*

| **Latent class marginal probabilities** | | | | | |
| --- | --- | --- | --- | --- | --- |
|  |  | Margin | SE | [95% conf. interval] | |
|  | Class |  |  |  |  |
|  | 1 | .3497347 | .1412803 | .1373125 | .6450572 |
|  | 2 | .1717081 | .0286273 | .1225957 | .2352207 |
|  | 3 | .4785572 | .1423216 | .2308247 | .7373059 |

| **Latent class marginal means** | | | | | |
| --- | --- | --- | --- | --- | --- |
| Class |  | Margin | SE | [95% conf. interval] | |
| 1 |  |  |  |  |  |
|  | CRT 24/7 | .7904795 | .3113643 | .0865219 | .9933897 |
|  | Crisis assessment | .2416834 | .0625361 | .1403665 | .3835055 |
|  | Crisis telephone | 1 | . | . | . |
|  | Crisis café | .0572859 | .0327774 | .0181585 | .1664322 |
|  | Crisis house | .4909557 | .1103987 | .2886449 | .696273 |
|  | Police St Triage | .5536568 | .0772712 | .4019382 | .6959974 |
|  | PDU | .6102218 | .1123555 | .3828194 | .7980397 |
| 2 |  |  |  |  |  |
|  | CRT 24/7 | .9999979 | .0008112 | 0 | 1 |
|  | Crisis assessment | .2749252 | .0816715 | .1451961 | .4584051 |
|  | Crisis telephone | 4.67e-06 | .0034645 | 0 | 1 |
|  | Crisis café | .1301439 | .0608066 | .0496211 | .3000771 |
|  | Crisis house | .1952177 | .0717182 | .0902028 | .3724426 |
|  | Police St Triage | .8339901 | .0679135 | .6576326 | .9292738 |
|  | PDU | .4708239 | .0926101 | .3004055 | .648327 |
| 3 |  |  |  |  |  |
|  | CRT 24/7 | 6.33e-07 | .000336 | 0 | 1 |
|  | Crisis assessment | .4835249 | .100892 | .297807 | .6739081 |
|  | Crisis telephone | .5921541 | .1269443 | .3413367 | .8026751 |
|  | Crisis café | .3064952 | .091354 | .1599064 | .5064529 |
|  | Crisis house | .2199214 | .0515855 | .135232 | .3369806 |
|  | Police St Triage | .6646179 | .0782262 | .4990252 | .7976681 |
|  | PDU | .474216 | .0633454 | .354076 | .5974173 |

| **Fit statistic** | **Value** |  |
| --- | --- | --- |
| Information criteria |  |  |
| AIC Akaike's information | 1554.780 |  |
| BIC Bayesian information | 1624.903 |  |

**CRT 24/7; Crisis assessment; Crisis tel; Crisis café; Crisis house; PDU; Police st triage; ADU (Missing= 35)**

2-Classes

Number of observations = 179

| **Latent class marginal probabilities** | | | | | |
| --- | --- | --- | --- | --- | --- |
|  |  | Margin | SE | [95% conf. interval] | |
|  | Class |  |  |  |  |
|  | 1 | 1 | - | - | - |
|  |  |  |  |  |  |

| **Latent class marginal means** | | | | | |
| --- | --- | --- | --- | --- | --- |
| Class |  | Margin | SE | [95% conf. interval] | |
| 1 |  |  |  |  |  |
|  | CRT 24/7 | .4476744 | .0379153 | .3750497 | .5226022 |
|  | Crisis assessment | .3631285 | .0359443 | .2959964 | .4360555 |
|  | Crisis telephone | .6327684 | .0362331 | .5593441 | .700509 |
|  | Crisis café | .1885714 | .0295695 | .1372784 | .2534005 |
|  | Crisis house | .3103448 | .0350723 | .246071 | .382881 |
|  | Police St Triage | .6551724 | .0360333 | .5815768 | .7220108 |
|  | PDU | .5202312 | .0379832 | .4458873 | .5936898 |
|  | ADU | .12 | .0245648 | .0795586 | .1770445 |
|  |  |  |  |  |  |

| **Fit statistic** | **Value** |  |
| --- | --- | --- |
| Information criteria |  |  |
| AIC Akaike's information | 1696.999 |  |
| BIC Bayesian information | 1722.498 |  |

2-Classes

Number of observations = 179

| **Latent class marginal probabilities** | | | | | |
| --- | --- | --- | --- | --- | --- |
|  |  | Margin | SE | [95% conf. interval] | |
|  | Class |  |  |  |  |
|  | 1 | .8115972 | .0295289 | .7468572 | .8628214 |
|  | 2 | .1884028 | .0295289 | .1371786 | .2531428 |

| **Latent class marginal means** | | | | | |
| --- | --- | --- | --- | --- | --- |
| Class |  | Margin | SE | [95% conf. interval] | |
| 1 |  |  |  |  |  |
|  | CRT 24/7 | .505255 | .0425268 | .4225241 | .5876992 |
|  | Crisis assessment | .3485278 | .0396166 | .275381 | .4295853 |
|  | Crisis telephone | .6650051 | .0394697 | .5838076 | .7374845 |
|  | Crisis café | 2.55e-08 | .0000498 | 0 | 1 |
|  | Crisis house | .2980586 | .0385372 | .2283635 | .3785884 |
|  | Police St Triage | .6467622 | .0401998 | .5646274 | .7210571 |
|  | PDU | .4707513 | .0420047 | .3899386 | .5531269 |
|  | ADU | .091591 | .0242143 | .0539229 | .1513625 |
| 2 |  |  |  |  |  |
|  | CRT 24/7 | .2092623 | .0704228 | .103075 | .3786599 |
|  | Crisis assessment | .426023 | .0858637 | .2716394 | .5963137 |
|  | Crisis telephone | .4958075 | .0868394 | .3323386 | .6601776 |
|  | Crisis café | .9999981 | .0012687 | 0 | 1 |
|  | Crisis house | .3626533 | .0836385 | .2187277 | .5362769 |
|  | Police St Triage | .6918443 | .0812342 | .5154823 | .8257157 |
|  | PDU | .7428447 | .0783277 | .5639359 | .8658162 |
|  | ADU | .2418015 | .0744663 | .125766 | .4141752 |

| **Fit statistic** | **Value** |  |
| --- | --- | --- |
| Information criteria |  |  |
| AIC Akaike's information | 1687.540 |  |
| BIC Bayesian information | 1741.726 |  |

3-Classes

Number of observations = 179 / *Convergence not achieved*

| **Latent class marginal probabilities** | | | | | |
| --- | --- | --- | --- | --- | --- |
|  |  | Margin | SE | [95% conf. interval] | |
|  | Class |  |  |  |  |
|  | 1 | .4371196 | .1076089 | .2478605 | .6466476 |
|  | 2 | .3743038 | .1072189 | .1960631 | .5947146 |
|  | 3 | .1885766 | .0295467 | .1373179 | .2533501 |

| **Latent class marginal means** | | | | | |
| --- | --- | --- | --- | --- | --- |
| Class |  | Margin | SE | [95% conf. interval] | |
| 1 |  |  |  |  |  |
|  | CRT 24/7 | .5010657 | .0709305 | .3654413 | .6365335 |
|  | Crisis assessment | .2597543 | .0731149 | .1427641 | .4250753 |
|  | Crisis telephone | 1 | .0000852 | 0 | 1 |
|  | Crisis café | 2.45e-08 | .0000626 | 0 | 1 |
|  | Crisis house | .4214835 | .0800627 | .2768309 | .5809959 |
|  | Police St Triage | .531965 | .0874433 | .363473 | .6934697 |
|  | PDU | .5392246 | .0725666 | .3976627 | .6747298 |
|  | ADU | .1503096 | .0503976 | .0754674 | .2771259 |
| 2 |  |  |  |  |  |
|  | CRT 24/7 | .5107814 | .0818488 | .354607 | .664879 |
|  | Crisis assessment | .4517722 | .0699698 | .321428 | .5890869 |
|  | Crisis telephone | .2805641 | .2007414 | .052606 | .7325412 |
|  | Crisis café | 1.52e-08 | . | . | . |
|  | Crisis house | .1543243 | .0544288 | .0745723 | .2924174 |
|  | Police St Triage | .779048 | .0593104 | .6421702 | .8738523 |
|  | PDU | .392532 | .0683724 | .2692022 | .5312869 |
|  | ADU | .0249556 | .0243418 | .0035898 | .1538529 |
| 3 |  |  |  |  |  |
|  | CRT 24/7 | .2088314 | .070307 | .1028442 | .3780217 |
|  | Crisis assessment | .4267941 | .0858442 | .2723579 | .596958 |
|  | Crisis telephone | .4964945 | .0867945 | .3330301 | .6607117 |
|  | Crisis café | .9999995 | .0004497 | 0 | 1 |
|  | Crisis house | .3624966 | .0836177 | .2186194 | .5360972 |
|  | Police St Triage | .6924601 | .0811211 | .5162567 | .8261009 |
|  | PDU | .7427761 | .0783144 | .5639155 | .8657424 |
|  | ADU | .2416173 | .0744307 | .125652 | .4139394 |

| **Fit statistic** | **Value** |  |
| --- | --- | --- |
| Information criteria |  |  |
| AIC Akaike's information | 1681.458 |  |
| BIC Bayesian information | 1761.142 |  |

**CRT 24/7; Crisis assessment; Crisis tel; Crisis café; Crisis house; PDU; Police st triage; ADU; Ambulance st triage (Missing= 35)**

1-Class

Number of observations = 179

| **Latent class marginal probabilities** | | | | | |
| --- | --- | --- | --- | --- | --- |
|  |  | Margin | SE | [95% conf. interval] | |
|  | Class |  |  |  |  |
|  | 1 | 1 | - | - | - |
|  |  |  |  |  |  |

| **Latent class marginal means** | | | | | |
| --- | --- | --- | --- | --- | --- |
| Class |  | Margin | SE | [95% conf. interval] | |
| 1 |  |  |  |  |  |
|  | CRT 24/7 | .4476744 | .0379153 | .3750497 | .5226022 |
|  | Crisis assessment | .3631285 | .0359443 | .2959964 | .4360555 |
|  | Crisis telephone | .6327684 | .0362331 | .5593441 | .700509 |
|  | Crisis café | .1885714 | .0295695 | .1372784 | .2534005 |
|  | Crisis house | .3103448 | .0350723 | .246071 | .382881 |
|  | Police St Triage | .6551724 | .0360333 | .5815768 | .7220108 |
|  | Ambulance st Triage | .2 | .0302372 | .147205 | .2658276 |
|  | PDU | .5202312 | .0379832 | .4458873 | .5936898 |
|  | ADU | .12 | .0245648 | .0795586 | .1770445 |
|  |  |  |  |  |  |

| **Fit statistic** | **Value** |  |
| --- | --- | --- |
| Information criteria |  |  |
| AIC Akaike's information | 1696.999 |  |
| BIC Bayesian information | 1722.498 |  |

2-Classes

Number of observations = 179 / *Convergence not achieved*

| **Latent class marginal probabilities** | | | | | |
| --- | --- | --- | --- | --- | --- |
|  |  | Margin | SE | [95% conf. interval] | |
|  | Class |  |  |  |  |
|  | 1 | .8000322 | .0302202 | .7342436 | .8527998 |
|  | 2 | .1999678 | .0302202 | .1472002 | .2657564 |

| **Latent class marginal means** | | | | | |
| --- | --- | --- | --- | --- | --- |
| Class |  | Margin | SE | [95% conf. interval] | |
| 1 |  |  |  |  |  |
|  | CRT 24/7 | .5127758 | .0428376 | .4292416 | .5956023 |
|  | Crisis assessment | .3535208 | .0400398 | .2794935 | .4353091 |
|  | Crisis telephone | .6600588 | .0399089 | .5780938 | .7334426 |
|  | Crisis café | 2.80e-08 | .0000141 | 0 | 1 |
|  | Crisis house | .288003 | .0384301 | .2188428 | .3687034 |
|  | Police St Triage | .641606 | .0406267 | .5587421 | .7167961 |
|  | Ambulance st Triage | 1.52e-08 | . | . | . |
|  | PDU | .4775611 | .0423435 | .39593 | .5604087 |
|  | ADU | .0786122 | .0227557 | .0440615 | .1363902 |
| 2 |  |  |  |  |  |
|  | CRT 24/7 | .1972338 | .0668749 | .096957 | .3598893 |
|  | Crisis assessment | .4015654 | .0826275 | .2548607 | .5683102 |
|  | Crisis telephone | .5251245 | .0841173 | .3634193 | .6817269 |
|  | Crisis café | .9428596 | .0392339 | .798363 | .9856665 |
|  | Crisis house | .3987203 | .0827102 | .2521874 | .5659584 |
|  | Police St Triage | .7100577 | .0774207 | .5395692 | .8365419 |
|  | Ambulance st Triage | .9999999 | .0000472 | 0 | 1 |
|  | PDU | .6980177 | .0797402 | .5240897 | .829108 |
|  | ADU | .284774 | .0762063 | .1605386 | .4532422 |

| **Fit statistic** | **Value** |  |
| --- | --- | --- |
| Information criteria |  |  |
| AIC Akaike's information | 1706.691 |  |
| BIC Bayesian information | 1764.064 |  |

3-Classes

Number of observations = 179 / *Convergence not achieved*

| **Latent class marginal probabilities** | | | | | |
| --- | --- | --- | --- | --- | --- |
|  |  | Margin | SE | [95% conf. interval] | |
|  | Class |  |  |  |  |
|  | 1 | .3981152 | .1037747 | .2206043 | .607186 |
|  | 2 | .4015615 | .1037765 | .2235039 | .6100296 |
|  | 3 | .2003233 | .0302489 | .147497 | .2661624 |

| **Latent class marginal means** | | | | | |
| --- | --- | --- | --- | --- | --- |
| Class |  | Margin | SE | [95% conf. interval] | |
| 1 |  |  |  |  |  |
|  | CRT 24/7 | .5278505 | .0801048 | .3732154 | .6773199 |
|  | Crisis assessment | .2556681 | .0729384 | .1394559 | .4213102 |
|  | Crisis telephone | 1 | .0000551 | 0 | 1 |
|  | Crisis café | 2.90e-08 | .0000205 | 0 | 1 |
|  | Crisis house | .4180234 | .0817109 | .2710721 | .5811278 |
|  | Police St Triage | .498484 | .096214 | .3185649 | .6787967 |
|  | Ambulance st Triage | 1.52e-08 | . | . | . |
|  | PDU | .5695377 | .0823131 | .4065888 | .7186987 |
|  | ADU | .1312914 | .0485755 | .0615525 | .2582965 |
| 2 |  |  |  |  |  |
|  | CRT 24/7 | .4979079 | .0784962 | .3489227 | .6472655 |
|  | Crisis assessment | .4500755 | .0692075 | .3211685 | .5860537 |
|  | Crisis telephone | .3281836 | .1702748 | .0970815 | .6893875 |
|  | Crisis café | 1.22e-07 | .0000415 | 4.7e-297 | 1 |
|  | Crisis house | .1591953 | .056783 | .0761611 | .30306 |
|  | Police St Triage | .7814861 | .0586717 | .645872 | .8752014 |
|  | Ambulance st Triage | 2.54e-08 | .0000192 | 0 | 1 |
|  | PDU | .3882223 | .0672003 | .2671077 | .5249206 |
|  | ADU | .027781 | .0268342 | .00406 | .1668717 |
| 3 |  |  |  |  |  |
|  | CRT 24/7 | .1968299 | .0667593 | .096747 | .3592665 |
|  | Crisis assessment | .4023584 | .0826122 | .2555944 | .5689818 |
|  | Crisis telephone | .5258719 | .0840603 | .3641932 | .6823018 |
|  | Crisis café | .9428572 | .0392347 | .7983602 | .9856654 |
|  | Crisis house | .3984402 | .0826867 | .2519731 | .5656634 |
|  | Police St Triage | .7106791 | .0773007 | .5403709 | .8369262 |
|  | Ambulance st Triage | 1 | .0000295 | 0 | 1 |
|  | PDU | .6979767 | .0797106 | .5241241 | .8290333 |
|  | ADU | .2846001 | .0761742 | .1604289 | .4530209 |

| **Fit statistic** | **Value** |  |
| --- | --- | --- |
| Information criteria |  |  |
| AIC Akaike's information | 1706.160 |  |
| BIC Bayesian information | 1795.407 |  |

**CRT 24/7; Crisis assessment; Crisis tel; Crisis café; Crisis house; PDU; Police st triage; ADU; Ambulance st triage; Integrated management; Shared staffing (Missing= 40)**

1-Class

Number of observations = 179

| **Latent class marginal probabilities** | | | | | |
| --- | --- | --- | --- | --- | --- |
|  |  | Margin | SE | [95% conf. interval] | |
|  | Class |  |  |  |  |
|  | 1 | 1 | - | - | - |
|  |  |  |  |  |  |

| **Latent class marginal means** | | | | | |
| --- | --- | --- | --- | --- | --- |
| Class |  | Margin | SE | [95% conf. interval] | |
| 1 |  |  |  |  |  |
|  | CRT 24/7 | .4476744 | .0379153 | .3750497 | .5226022 |
|  | Crisis assessment | .3631285 | .0359443 | .2959964 | .4360555 |
|  | Crisis telephone | .6327684 | .0362331 | .5593441 | .700509 |
|  | Crisis café | .1885714 | .0295695 | .1372784 | .2534005 |
|  | Crisis house | .3103448 | .0350723 | .246071 | .382881 |
|  | Police St Triage | .6551724 | .0360333 | .5815768 | .7220108 |
|  | Ambulance st Triage | .2 | .0302372 | .147205 | .2658276 |
|  | PDU | .5202312 | .0379832 | .4458873 | .5936898 |
|  | ADU | .12 | .0245648 | .0795586 | .1770445 |
|  | Integrated management | .183908 | .0293694 | .1331229 | .2485132 |
|  | Shared Staffing | .747191 | .0325763 | .6782365 | .8056038 |
|  |  |  |  |  |  |

| **Fit statistic** | **Value** |  |
| --- | --- | --- |
| Information criteria |  |  |
| AIC Akaike's information | 2245.511 |  |
| BIC Bayesian information | 2280.572 |  |

2-Classes

Number of observations = 179 / *Convergence not achieved*

| **Latent class marginal probabilities** | | | | | |
| --- | --- | --- | --- | --- | --- |
|  |  | Margin | SE | [95% conf. interval] | |
|  | Class |  |  |  |  |
|  | 1 | .7996381 | .0302704 | .7337496 | .852499 |
|  | 2 | .2003619 | .0302704 | .147501 | .2662504 |

| **Latent class marginal means** | | | | | |
| --- | --- | --- | --- | --- | --- |
| Class |  | Margin | SE | [95% conf. interval] | |
| 1 |  |  |  |  |  |
|  | CRT 24/7 | .5128017 | .0428381 | .4292659 | .5956285 |
|  | Crisis assessment | .3536884 | .0400587 | .2796234 | .4355111 |
|  | Crisis telephone | .6598999 | .0399272 | .5779014 | .7333199 |
|  | Crisis café | 1.52e-08 | . | . | . |
|  | Crisis house | .2880285 | .0384312 | .2188656 | .3687304 |
|  | Police St Triage | .6416144 | .0406268 | .5587501 | .7168045 |
|  | Ambulance st Triage | 3.89e-08 | .000021 | 0 | 1 |
|  | PDU | .4775563 | .0423437 | .395925 | .5604045 |
|  | ADU | .0786136 | .022756 | .0440625 | .136392 |
|  | Integrated management | .1940792 | .0335489 | .1365657 | .2682878 |
|  | Shared Staffing | .7564299 | .0361456 | .6788937 | .8202021 |
| 2 |  |  |  |  |  |
|  | CRT 24/7 | .197259 | .0668782 | .0969743 | .3599171 |
|  | Crisis assessment | .4008189 | .08257 | .2542817 | .567534 |
|  | Crisis telephone | .5260062 | .0840826 | .3642732 | .6824605 |
|  | Crisis café | .9428549 | .0392354 | .7983576 | .9856645 |
|  | Crisis house | .3986854 | .0827089 | .2521582 | .5659249 |
|  | Police St Triage | .7100296 | .0774236 | .5395391 | .8365211 |
|  | Ambulance st Triage | .9999998 | .000072 | 0 | 1 |
|  | PDU | .6980296 | .0797396 | .5241009 | .8291176 |
|  | ADU | .2847385 | .0762032 | .1605117 | .4532052 |
|  | Integrated management | .1433584 | .0594435 | .0608608 | .3017518 |
|  | Shared Staffing | .7106276 | .0767011 | .5417471 | .8360998 |

| **Fit statistic** | **Value** |  |
| --- | --- | --- |
| Information criteria |  |  |
| AIC Akaike's information | 2081.268 |  |
| BIC Bayesian information | 2151.391 |  |

3-Classes

Number of observations = 179 / *Convergence not achieved*

| **Latent class marginal probabilities** | | | | | |
| --- | --- | --- | --- | --- | --- |
|  |  | Margin | SE | [95% conf. interval] | |
|  | Class |  |  |  |  |
|  | 1 | .597152 | .0636998 | .4686963 | .7135326 |
|  | 2 | .2026422 | .0603421 | .1089202 | .3457203 |
|  | 3 | .2002058 | .0302093 | .1474452 | .2659563 |

| **Latent class marginal means** | | | | | |
| --- | --- | --- | --- | --- | --- |
| Class |  | Margin | SE | [95% conf. interval] | |
| 1 |  |  |  |  |  |
|  | CRT 24/7 | .5462907 | .0534898 | .4409559 | .6476379 |
|  | Crisis assessment | .1330753 | .0823166 | .0365261 | .3833022 |
|  | Crisis telephone | .704502 | .0479001 | .6029708 | .7891483 |
|  | Crisis café | 5.04e-09 | . | . | . |
|  | Crisis house | .2632246 | .0446119 | .1854077 | .3592962 |
|  | Police St Triage | .6072577 | .0531245 | .4998052 | .7052403 |
|  | Ambulance st Triage | 1.12e-08 | . | . | . |
|  | PDU | .4894719 | .0515899 | .3901324 | .5896499 |
|  | ADU | .0673904 | .0252965 | .0317879 | .1372164 |
|  | Integrated management | 1.52e-08 | . | . | . |
|  | Shared Staffing | .6716165 | .0541541 | .5582959 | .7679482 |
| 2 |  |  |  |  |  |
|  | CRT 24/7 | .4166968 | .0915868 | .254477 | .599211 |
|  | Crisis assessment | .9999999 | .000063 | 0 | 1 |
|  | Crisis telephone | .5311984 | .1249751 | .2976193 | .7518629 |
|  | Crisis café | 6.42e-09 | . | . | . |
|  | Crisis house | .3600032 | .0878507 | .2103736 | .5428879 |
|  | Police St Triage | .7380715 | .0815651 | .5520692 | .865635 |
|  | Ambulance st Triage | 1.21e-08 | . | . | . |
|  | PDU | .4417826 | .0900063 | .2790327 | .6180793 |
|  | ADU | .1110484 | .0593663 | .0370133 | .2887645 |
|  | Integrated management | .7443697 | .2054249 | .2597788 | .9602556 |
|  | Shared Staffing | 1 | .0000466 | 0 | 1 |
| 3 |  |  |  |  |  |
|  | CRT 24/7 | .1965768 | .0666809 | .0966217 | .3588593 |
|  | Crisis assessment | .4046925 | .0826788 | .2575809 | .5711829 |
|  | Crisis telephone | .5256193 | .0840194 | .3640411 | .6820049 |
|  | Crisis café | .9428572 | .0392347 | .7983603 | .9856654 |
|  | Crisis house | .3987826 | .0827161 | .252234 | .5660253 |
|  | Police St Triage | .7110619 | .0772131 | .5408973 | .8371452 |
|  | Ambulance st Triage | 1 | .0000253 | 0 | 1 |
|  | PDU | .6992859 | .0795025 | .5257014 | .8298978 |
|  | ADU | .2848447 | .0762167 | .1605868 | .4533257 |
|  | Integrated management | .1435289 | .0594859 | .0609551 | .3019892 |

| **Fit statistic** | **Value** |  |
| --- | --- | --- |
| Information criteria |  |  |
| AIC Akaike's information | 2002.417 |  |
| BIC Bayesian information | 2098.038 |  |
